# Awareness and Attitudes of Student Dietitians in Lebanon, UK and USA towards Food Safety

**DOI:** 10.3390/foods10081875

**Published:** 2021-08-13

**Authors:** Ellen W. Evans, Elizabeth C. Redmond, Nisreen Alwan, Sanja Ilic

**Affiliations:** 1 ZERO2FIVE Food Industry Centre, Cardiff Metropolitan University, Western Avenue, Llandaff, Cardiff CF5 2YB, Wales, UK; elevans@cardiffmet.ac.uk (E.W.E.); eredmond@cardiffmet.ac.uk (E.C.R.); 2 College of Health Sciences, Abu Dhabi University, Abu Dhabi P.O. Box 59911, United Arab Emirates; nisreen.alwan@adu.ac.ae; 3 School of Health Sciences, Modern University for Business and Science, Beirut P.O. Box 113 7501, Lebanon; 4 Department of Human Sciences, Ohio State University, Columbus, OH 43210, USA

**Keywords:** dietitians, food safety, food safety knowledge, attitudes, patient education

## Abstract

Allied health professionals such as dietitians can play a critical role in providing food safety advice to vulnerable consumers. To maximize food-related health and wellbeing, food needs to be safe and nutritious; consequently, food safety is referred to in international curricula for the training of dietitians. The purpose of this study was to explore the awareness and attitudes of student dietitians from three international institutions towards food safety. A total of 207 student dietitians participated in the study from Columbus, OH, USA (*n* = 99), Cardiff, Wales, UK (*n* = 78) and Beirut, Lebanon (*n* = 30). Completion of the study established that the students in three dietetic training programs lacked awareness of key food safety concepts. Close to half (43%) were not familiar with Campylobacter, with the USA students being significantly less knowledgeable (*p* < 0.001) with 58% being unaware of the pathogen. Understanding of safe handling of leftovers was the lowest for the students in all institutions; only 46% described appropriate reheating practices, with significantly lower (*p* < 0.001) understanding in Lebanon (28%). The students reported a good understanding of vulnerable populations and perceived food safety to be important for these groups. However, the knowledge of certain high-risk foods was lacking. For instance, 69% of students thought that fresh squeezed juices and smoothies made with raw fruits and vegetables were safe for vulnerable patients, with the UK students being the least familiar with this risk (16%). This is the first study of its kind to take an international perspective of student dietitian food safety awareness and attitudes; the findings are important to dietetic food safety educators and recommendations are made to further explore the interpretation of food safety requirements in international dietetic curricula. Future studies should extend student dietetic research to address attitudes, self-efficacy and the overall readiness to deliver food safety advice to the patients and the community.

## 1. Introduction

Among consumers in Europe and the US, healthcare professionals, such as dietitians, are the most trusted [1,2] and are a preferred source for food safety information [3,4]. Vulnerable consumers such as those living with HIV [4], individuals living with cancer [5], people receiving chemotherapy treatment [6] and transplant patients [7] reportedly prefer verbal communication from healthcare professionals including dietitians for delivery of food safety advice. This is particularly the case with older adults, who are more likely to seek information from family physicians and dietitians [8]. It has been shown that high risk patients, such as those receiving chemotherapy treatment are often not aware of their increased susceptibility to foodborne diseases [9]. Trust in the source of food safety information is a critical element for the successful delivery of food safety information. Indeed, the impact and effectiveness of food safety information is largely dependent upon the perceived reliability of the source [10]. Trust in the information provider is reported to be important for consumers when receiving food safety information [1,10].

It is well documented that there are increased risks of foodborne infection for individuals who have compromised immune systems [11]. For example, people with diabetes have an increased risk of foodborne illness due to autonomic neuropathy, poor glycemic control and reduced gastric acid production [12], whereas with people living with cancer, the chemotherapeutic agents (chemotherapy) used for the treatment of cancer causes diminution of the host immune response which increases susceptibility of patients to opportunistic pathogens [13]. As allied health professionals within a broader multidisciplinary healthcare team, dietitians may have access to many of these patient groups and individuals who are at risk of foodborne illness and are therefore well placed to deliver food-safety information to reduce the risk of foodborne illness in vulnerable patients [14].

Targeted food safety information for at-risk populations may help to prevent cases of foodborne infections [11,15,16]. Indeed, if vulnerable individuals received food safety information from adequately trained, credible healthcare professionals such as dietitians the importance of food safety could be emphasized [14]. However, previous research has determined gaps in general food safety knowledge and pathogen awareness of practicing registered dietitians [17,18]. Furthermore, it is reported that dietitians only occasionally provide food safety advice to vulnerable consumers [19,20]. Cumulatively, these factors may result in patients being inadequately informed and thus potentially more susceptible to foodborne illness.

It is accepted that food needs to be safe and nutritious in order to maximize food-related health and wellbeing, and therefore, food safety is included in international curricula for the training of dietitians. For example, in the USA, the Accreditation Council for Education in Nutrition and Dietetics states that dietetic graduates should be able to “describe safety principles related to food, personnel and consumers” [21]. Similarly, in the UK, the British Dietetic Association Curriculum Framework states that dietetic graduates

*“must have applied knowledge of food safety legislation and practice to manage and evaluate the service of safe food as well as a broad knowledge of structure and function of common microbes which cause infection and disease”*

[22] (British Dietetic Association, 2013)

Both such examples illustrate that dietetic graduate should understand foodservice food safety principles, food safety legislation and microbiology. However, there is a lack of emphasis on the ability of dietetic graduates to identify vulnerable groups at risk of foodborne illness, to educate such groups regarding the associated vulnerability and coach food safety practices that are essential to minimize associated risks.

While the food safety information is currently presented solely in the context of foodservice management and not an integral part of dietetic training, the actual level of future dietitians’ understanding of food safety is largely unknown. The current level of food safety knowledge among dietetics students is largely unknown. The International Network of Dietetic Food Safety Educators has published the statement about the need for to better understand trainee dietitians’ food safety knowledge, training experiences and attitudes toward the delivery of food safety information [14,23]. This is of particular importance when considering the future needs of dietetics programs and evolving requirements of dietetics profession expanding from mostly focusing on clinical practice and institutional foodservice management, to the role in counselling and providing food and diet advice to the communities outside of the health care institutions

Previous research has identified gaps in the food safety knowledge of dietitians; consequently, the purpose of this study was to determine and evaluate food safety awareness and associated attitudes of student dietitians from three international institutions.

## 2. Materials and Methods

### 2.1. Data Collection Tool

A self-complete questionnaire was designed and developed based upon a thorough review of literature which determined key food safety practices of importance for consumers and vulnerable patient groups. The questionnaire was designed to determine the knowledge of dietetic students in relation to key food safety areas, namely, foodborne pathogens, cooking, refrigeration, storage, hand washing, cross-contamination, vulnerable groups associated with increased risk of foodborne infection and locations associated with foodborne infection. Most knowledge-based questions were multiple choice. Positively and negatively worded attitudinal statements were developed in relation to key food safety areas, and a five-point Likert-type scale was utilized for responses.

Piloting was undertaken by student dietitians Cardiff Metropolitan University (*n* = 10), after which, some questions were removed; the clarity of wording to some questions was amended, and the length, formatting and layout of the paper-based questionnaire was amended. The online version of the questionnaire was created and managed in Qualtrics (Qualtrics.XM, Provo, UT, USA).

### 2.2. Data Collection

Students (aged > 18 years) enrolled on accredited dietetic programs, studying at Cardiff Metropolitan University, Wales, UK; the Modern University for Business and Science, Beirut, Lebanon; and the Ohio State University, Columbus, OH, USA, were recruited for participation in the study. Students were provided with a participant information sheet and were required to provide consent for participation. A paper-based questionnaire was made available for completion in UK and Lebanon, the questionnaire was distributed to dietetic students following a lecture. The questionnaire was administered to USA dietetic students digitally to complete in their own time.

### 2.3. Statistical Analysis

The dataset of online completed responses was downloaded as a Microsoft Excel 2010 (Microsoft; Redmond, WA, USA) spreadsheet and paper-based responses were manually entered to the dataset. All responses were coded and only identifiable by an assigned identification number for each participant. All participants that completed less than 85% of the questionnaire were removed from the data set. Use was made of Microsoft Excel (Microsoft; Redmond, WA, USA). Perceptions of risks were scored on the ten-point scale and scores ranked based on weighted averages [24]. Likert scale responses were analyzed by weighted averages using frequencies as weights and used for ranking (ten Klooster et al., 2008). Weighted averages were calculated as the sum of the products for each response divided by all the participants who committed an answer (on a scale of 1–10), divided by the total number of respondents [25]. The weighted averages, upper and lower quartiles were presented in tables. Weighted averages were calculated as the sum of the products for each response divided by all the participants who committed an answer (on a scale of 1–10), divided by the total number of respondents [25]. The differences between the student responses from the three programs and the effect of received food safety education on the knowledge were tested using chi square and significant findings presented tables. All statistical analyses were conducted using SPSS Statistics 20 (IBM^®^ Software Group; Chicago, IL, USA).

### 2.4. Ethical Approval

All methods and materials in the study were granted Ethical approval from Cardiff Metropolitan University School of Health Sciences Research Ethics Committee (Project reference number: (reference no: 9299). The study was approved by The Ohio State Institutional Review Board (2018E0032) and by the Ethics committees at the Modern University for Business and Science (MU-20180701).

## 3. Results

### 3.1. Respondent Demographic Characteristics

A total of 207 student dietitians participated in the study. As illustrated in Table 1, of the student dietitians who participated in the study, most (87%) were aged 18–29 years and female (89%). The largest group of dietetic students were in Columbus, Ohio, USA (*n* = 99), 78 were in Cardiff, Wales, UK and 30 were in Beirut, Lebanon. The majority studied full time (88%) and were in the second (35%) or third year (38%) of their dietetic studies. Over three-quarters (78%) reported having completed food safety training or education as part of their degree; all participating students from the UK and Lebanon reported completing such training, compared with only 54% of participating students from the USA. Analysis is conducted according to location and reported receipt of food safety education.

### 3.2. Pathogen Awareness

It was determined that the majority of dietetic students reported awareness of *Escherichia coli* (94%) and *Salmonella* (96%). Significant differences were determined in awareness of other pathogens according to location and reported receipt of previous food safety education. Students that reported receiving food safety education indicated significantly greater awareness of *Listeria, Campylobacter, Staphylococcus* and *Clostridium* (*p* < 0.005) (Table 2). UK dietetic students indicated greater awareness of *Campylobacter*, *Staphylococcus* and *Clostridium* than USA dietetic students (*p* < 0.05) (Table 3).

### 3.3. Cooking

In relation to cooking practices, there was vast awareness (91%) of the need to use a thermometer to check the temperature when cooking meat or poultry to ensure that it is safe to eat. No significant differences (*p* > 0.05) were determined according to country or according to education and awareness of the need to use a thermometer (Table 2). Statistically significant differences were determined (*p* < 0.001) in relation to other practices used to determine adequate cooking with higher proportions of UK students indicating awareness of two practices that are promoted by the UK Food Standards Agency (2018) to ensure that the ‘center is piping hot’ (78%) and to ‘pierce thickest part to ensure juices run clear’ (73%) (Table 3).

Almost all dietetic students (93%) agreed that inadequate cooking of food increases the risk of foodborne illness to vulnerable patient groups, and 71% thought that the meat thermometer use was important to determine food safety of cooked meats.

### 3.4. Refrigeration Practices

Only 67% of all dietetic students were aware of the need to use a thermometer to check the operating temperature of the refrigerator. Over three-quarters (73%) reported awareness of the recommended temperature a refrigerator should operate at to ensure food safety, and 77% stated the correct temperature. A significant difference (*p* < 0.001) was determined where 97% of students in Lebanon, compared to 88% in the UK and 63% in the USA, were familiar with the recommended temperature for safe refrigeration (Table 3). Similarly, significant differences (*p* < 0.001) were determined according to education, whereby 82% of those that had received food safety education perceived that they knew the recommended temperature, and 89% stated the correct temperature, compared to 40% and 38% of those that did not receive food safety education (Table 2).

Significant differences (*p* < 0.05) were determined between programs according to awareness of refrigeration practices, with 40% of USA students perceiving that the leftovers should be refrigerated immediately after cooking (compared to 4% UK and 20% Lebanon students). Only 32% of USA students were aware of the need to allow left-over cooked food to cool outside of the refrigerator before refrigerating (compared to 88% UK and 60% Lebanon students) (Table 3).

Accordingly, most dietetics students (90%) in UK and Lebanon indicated that it was important to maintain refrigerator temperatures below 5 °C for food safety, as well as know the actual temperature of the fridge (93%), as opposed to only determine if the fridge feels cold.

### 3.5. Storage Duration and Dealing with Leftovers

Although 70% of all dietetic students were aware that the ‘use by’ date is an indicator of food safety, a significant difference in awareness was determined regarding which date label is the best indicator of food safety. The majority of UK students (81%) and USA students (75%) were aware that it was the ‘use by’ date, whereas only 30% of students in Lebanon were aware; 40% of students in Lebanon perceived it to be the ‘best before’ date (X^2^ (10, *n* = 195) = 61.505, *p* = 0.000, Cramer’s V = 0.394). Although no significant differences were determined according to education (*p* > 0.05) (Table 2), significant differences (*p* < 0.05) regarding awareness of recommended storage durations were determined according to country (Table 3).

An outcome of interest is that less than half of student dietitians from UK, USA and Lebanon were aware of the recommended storage duration for RTE foods such as cooked sliced meat or smoked fish. This was reflected in predominantly neutral attitudes toward storage practices for food safety. Almost half of the students in all three programs (49%) were uncertain if the food that has been opened for longer than two days and kept refrigerated was still safe for vulnerable patients to consume. However, the majority (84%) understood that cold cuts (i.e., cooked ham) stored in the fridge were not safe to eat after ‘use-by’ date.

### 3.6. Hand Washing

Most student dietitians (77–98%) were aware of occasions that would require implementation of hand washing. Awareness of critical occasions such as washing hands after handling raw meat and poultry was reported by the majority with no significant differences according to country (95—100% in Lebanon and USA respectively). Awareness of when hands should be washed did not differ according to education; however, significant differences (*p* < 0.001) were determined between the countries, with all USA students reporting awareness of the need for hand washing before commencing food preparation compared to just 80% of dietetic students from Lebanon. Conversely, only 51% of USA students reported awareness of the need to implement hand washing after feeding or touching pets compared to 97% of UK and Lebanon students (*p* < 0.001) (Table 3).

While many of the dietetic students (78%) knew that it was not safe for people that have diarrhea to prepare food for vulnerable patients even if they wash their hands first, students from Lebanon indicated significantly lower understanding of this concept (X^2^ (10, *n* = 198) = 38.801, *p* = 0.000, wgt. average 3.35, (Table 4), with almost half of such students (43%) believing this was an acceptable food safety practice.

Similarly, more dietetic students from Lebanon believed that it was safe to rinse hands quickly after preparing raw meat if hands were washed thoroughly before starting food preparation in comparing to UK students (24% vs. 5%, respectively: X^2^ (4, *n* = 195) = 16.508, *p* = 0.002). This reflected overall lower attitude toward hand washing among Lebanese students, who on more occasions thought that rubbing hands and between fingers with soap and lather for 20 s before rinsing with hot water is too much for people to do at home, comparing to UK students (44% vs. 24%, respectively, X^2^ (4, *n* = 195) = 18.132, *p* = 0.001).

### 3.7. Cross-Contamination

Although many students were aware of domestic food handling practices that increase the risk of cross-contamination in the home, significant differences were determined according to food safety education and according to country. Awareness of all practices that increase the risk of cross-contamination were significantly greater among those that reported receiving food safety education (*p* < 0.05) (Table 2). Awareness of failing to wash hands after handling raw meat and before handling ready-to-eat food and washing raw poultry increasing the risk of cross-contamination was greatest among UK students (*p* < 0.05) (Table 3).

In general, dietetic students had positive attitudes towards prevention of cross-contamination. The majority (90%) of students in UK and Lebanon (Table 5) thought that it was good to wash the utensils and countertop with hot, soapy water after cutting raw meat or chicken and before continuing cooking, and 79% believed that it was important to avoid storing raw meat above ready-to-eat food in the fridge. However, the students in Lebanon were less familiar with the cross-contamination concept from washing raw chicken than UK students (X^2^ (4, *n* = 103) = 20.012 *p* = 0.000), and just under half believed that the chicken should be washed before cooking (44.8%).

### 3.8. Vulnerable Groups Associated with Increased Risk of Foodborne Infection

In general, dietetics students in all three studied programs had a good understanding about the food safety risks associated with vulnerable populations. The majority (87%) agreed that it is more important to implement food safety practices when a patient is immunosuppressed. They perceived the people with compromised immune systems to be at overall highest risk from contracting foodborne illness. Students in the three programs ranked the general category of people with compromised immune system as the most vulnerable population (overall wgt. average of 9.60; in comparison to general population wgt. average of 4.63), followed by cancer patients receiving treatment (Table 4). The USA students perceived young children as more vulnerable (X^2^ (16, *n* = 206) = 60.725, *p* = 0.000) wgt. average 8.78) than UK and Lebanon counterparts (7.44, 7.41, respectively), but all other susceptible groups were perceived similarly by the students in the three programs. Students who had received food safety information or training as a part of their degree, ranked pregnant women X^2^ (7, *n* = 206) = 15.039, *p* = 0.036) and HIV patients (X^2^ (7, *n* = 126) = 18.433, *p* = 0.010) somewhat higher, than their counterparts who had not received food safety training; however, the ranking order was the same regardless the food safety education.

Although the overall understanding of importance of food safety for the populations at risk was perceived as important by the dietetic students in all three countries, one fifth (19%) of future dietitians across the three programs thought that the smell and taste of food was a reliable method of indicating that food was safe to eat reflecting the lack of understanding of general food safety principles. A total of 40% believed that throwing food only few days past its use by date was wasteful.

Understanding of high-risk foods for vulnerable populations varied by the food type. Most students (79%) across programs knew that it was not safe for people in high-risk groups to eat soft cheese made from unpasteurized milk, like Brie or Camembert. However, over two thirds (69%) thought that drinking juices and smoothies made with raw fruits and vegetables was safe for vulnerable patients. The students from the UK had a lower understanding of this concept than their peers in the USA and Lebanon with only 16% disagreeing with the statement that drinking juices and smoothies made with raw fruits was safe for vulnerable patients.

Less than one third of students (30%) thought that vulnerable patients should not consume leftovers, while the majority agreed that it was not acceptable to leave food at a lukewarm temperature for the later consumption (77%). The students in the USA had significantly lower understanding of adequate practices for handling leftovers (X^2^ (10, *n* = 200) = 40.228, *p* = 0.000) with only one half (50%) believing that reheating foods to room temperature was unacceptable practice (Table 5).

### 3.9. Locations Associated with Foodborne Infection

The perception of risks of foodborne diseases relative to the foodservice venue among dietetics students in the US, UK, and Lebanon was similar for take-away/fast-food restaurants, patients own homes and foods prepared by the caregivers. Fast foods were considered to be the most likely to cause the foodborne disease (wgt. average 3.99, Table 4). Lebanese students perceived the foods prepared by the patients at home to pose significantly higher risk for foodborne infections than students in UK and USA (X^2^ (10, *n* = 201) = 35.217, *p* = 0.001) The students in all three programs perceived hospital prepared foods as the least likely to cause foodborne diseases. Among them, the USA students had the least confidence in hospital foods (wgt. average 6.09, X^2^ (18, *n* = 202) = 28.904, *p* = 0.05). Further, the perception of risk related to hospital foods and cafeterias was altered when the student received food safety training during their program (X^2^ (9, *n* = 202) = 21.972, *p* = 0.009).

## 4. Discussion

For the first time, this study gives an international perspective of food safety knowledge among dietetics students. Although there have been previous reports that have included dietetics student food safety knowledge, the number of studies in the last two decades has been scarce. Nevertheless, such research tends to focus on comparing university students who study dietetics with those that do not study dietetics; with those that study dietetics often having greater understanding of food safety [26,27,28,29]. Several studies also report on food safety knowledge and practices of international university students [27,30,31,32,33,34,35], despite identifying knowledge differences according to factors such as degree topic, gender and frequency of cooking; many of these studies identify the need to improve the food safety knowledge and practices of university students. However, any evaluation of food safety knowledge among dietetics students should take into consideration that the expectations from future dietitians are to deliver food safety information by patients and the community at large [36], a role that goes beyond the foodservice management.

In this study, 89% of participants were female; this is representative of the gender profile of practicing dietitians internationally, whereby 84–96% are female; the lack of gender diversity in the profession is known [37]. This study found that the dietetics students in the three institutions had a good understanding of several food safety concepts, including awareness of *E. coli* and *Salmonella* as human pathogens that can often cause foodborne diseases. This was true even for the students that had not previous food safety training. Frequent media coverage of high-profile outbreaks linked to *E. coli* and *Salmonella*, especially in produce and chicken, might be a contributing factor [38,39,40,41,42]. The students’ food safety knowledge was insufficient in a number of food safety concepts, including important pathogens such as *Campylobacter* and *L. monocytogenes*. This is of concern because *Campylobacter* is one of the most common causes of diarrheal disease in the US, accounting for the proportion of hospitalization from foodborne illnesses than *Salmonella* [43]. Similarly, *L. monocytogenes* causes severe foodborne infections and is one of the leading causes of death [43].The previous findings among Registered Dietitians in the USA are similar, where most were aware of *Salmonella* spp. and *E. coli* O157:H7 and least aware of *Campylobacter jejuni* [18]. It must also be acknowledged that similar levels of pathogen awareness exist among the general population, whereby the majority of consumers report awareness of *Salmonella* and *E. coli*, and awareness of *Listeria* and *Campylobacter* are particularly low [44,45,46]. In our study, the USA students had lower knowledge of *Campylobacter* than the students from UK or Lebanon, and as mentioned, this is of concern given the commonality of the pathogen.

Appropriate cooking practices are critical during domestic food preparation [47], as inadequate heat treatment is often implicated in incidence of foodborne infection [48,49]. To ensure cooking adequacy, UK consumers are advised to cut the thickest part of meat/poultry to ensure that juices run clear and it is steaming hot and has no pink meat [50]. However, visual evaluation of internal color is not an accurate indicator of doneness [51] as internal temperature cannot be judged by color and appearance [52]. Visual inspection is not recommended for the consumers in the US. The use of the cooking thermometer is suggested as the most accurate assessment for achieving adequate cooking [53]; while it is published that 24 to 69% of consumers are aware that using a food thermometer is the best way to tell when meat has been cooked thoroughly [54], the practice is not implemented widely in the community [54,55,56] cooking thermometers are said to be more commonly used by consumers in the USA than in Europe [57]. Even so, the majority of dietetics students in our study were aware of recommended practices and expressed positive attitudes towards the importance of proper cooking and the use of a thermometer. Higher proportions of UK students indicated awareness of visual evaluation practices promoted by the UK Food Standards Agency to determine cooking adequacy. This is of concern given that visual evaluation is not an accurate assessment for cooking adequacy.

Ensuring safe operating temperatures of domestic refrigerators is essential to limit microbiological growth. UK food safety requirements for domestic storage of refrigerated foods is ≤5.0 °C (≤41 °F) (Food Standards Agency, 2020), whilst USA food safety requirements for domestic refrigeration is 40 °F (4.4 °C) (FDA, 2017). International consumer research indicate that the majority of domestic refrigerators operate at temperatures exceeding recommendations [58,59,60]. Such food safety malpractice can have significant implications for domestic food safety; laboratory re-enactment research has established that refrigeration practices, contrary to consumer recommendations, can increase growth of foodborne pathogens, thus increasing the potential for foodborne disease [61]. In this study, student dietitians had significantly higher awareness of recommended refrigeration practices after they received food safety training. These findings highlight the importance of food safety education in didactic programs in dietetics to ensure awareness of the basic food safety concepts and recommendations. Previous studies have shown similar results [26,27,28,29].

Handling leftovers was the topic that the dietetics students had the least understanding of in this study. This is of concern because the high-risk patients report low understanding of leftover handling [9,23] and would benefit of the advice by their dietitians. In fact, the leftover handling was identified as one of the major knowledge gaps among cancer patients receiving treatment in the USA [9] and the UK [23]. Preventing prolonged storage of RTE food products is vital, particularly among vulnerable patient groups.

‘Use-by’ dates on food products ensure that potentially dangerous levels are not exceeded between production and consumption [62]. It is essential that ‘use-by’ dates are adhered to by consumers, as organoleptic attributes are not reliable methods to determine food safety as pathogens can grow to potentially unsafe numbers without adverse effects on the sensory attributes of the food [63]. Gaps were identified in dietetic student awareness of safe storage duration labeling, with some indicating confusion between ‘use by’ and ‘best before’ dates; this was particularly the case in Lebanon.

It is well documented that hand washing is one of the most important practices for infection control and maximizing food safety through the prevention of cross-contamination. Although the majority of student dieticians were aware of occasions that required hand washing, some gaps were identified, particularly in relation to the appropriate method for hand washing. The dietetic students in this study had positive attitudes towards prevention of cross-contamination, and awareness was associated with receiving food safety education. However, dietetic students in Lebanon were less familiar with the potential risk of cross-contamination from washing raw chicken before cooking. The practice of washing raw meat and poultry is known to cause cross-contamination and transfer of pathogens in the domestic kitchen environment [23,64] and is not a recommended practice for consumers to implement [65]. Dietetics students in the three studied programs in the US, UK and Lebanon had overall good understanding of vulnerable population and high-risk groups of patients more susceptible to foodborne infections. This is to be expected, as many courses in dietetics program cover high-risk populations from various perspectives. Furthermore, the students correctly perceived food safety to be particularly important for these groups. This is in accordance with the expectation and the previous reports, where educators ranked food safety competences as very important or essential for future dietitians [36]. College students have previously failed to identify older adults as being vulnerable to foodborne infection [29].

## 5. Conclusions

For the first time, in this study, an international perspective of food safety knowledge among dietetics students has been determined. Food safety knowledge, attitudes and risk perceptions among dietetics students have been presented and contrasted in three different world regions.

Findings from this study may suggest the lack of understanding of some general food safety principles may pose serious challenges to readiness of dietetics students to provide appropriate food safety advice to high-risk patients and the broader community. Although the current food safety education of students in didactic programs in dietetics lead to improve risk perceptions and overall better understanding of food safety risks faced by vulnerable population, additional training is required to address the specific patients’ needs, particularly those that live with the increased susceptibilities from infections in communities.

Although McCabe-Sellers and Beattie (2004) suggest that dietetics professionals can update knowledge and practice through online resources to stay knowledgeable and prepared to meet the food safety needs of clients, nevertheless, it is essential that dietitians obtain sufficient food safety knowledge at degree level and acquire the ability to provide food safety advice and information to vulnerable patient groups [66]. Similarly, online continuing education courses can be a convenient and effective method to enhance knowledge about food safety issues of high-risk populations among dietetics professionals [67]; the importance of embedding food safety information and the ability to deliver food safety information to vulnerable patients during undergraduate training must not be overlooked. Giving time for the inclusion of food safety education in undergraduate teaching of dietitians may highlight the importance of delivering food safety education to patients. It has been established that health professionals currently perceive that they do not have enough time to provide food safety education to patients [68]. It has previously been suggested that there is a need to consider what role cooking skills could have in dietetics training as a professional competency for practice [69]; there is a need to consider if this could contribute to food safety education and communication.

It must be acknowledged that the three institutions surveyed in this study only delivered food safety education in the context of foodservice management; it is possible that other institutions interpret the curriculum differently and have a different approach to food safety education. Future research to explore the interpretation of curriculum requirements would give an overview of best practices in teaching food safety to dietetics students.

Despite having explored the attitudes and awareness of dietetic students as they relate to food safety risk perceptions in this study, the research has identified the need to study the perceptions of dietetic students towards the provision of food safety information and their perception of their role as food safety educators.

## Figures and Tables

**Table 1:** Demographic profile of dietetic student participants (*n* = 207).

Demographic Characteristics		*n*	%
Gender	Female	184	89%
	Male	21	10%
	not disclosed	2	1%
Age	18–29 years	180	87%
	30–44 years	23	11%
	>45 years	4	2%
Location	Cardiff, UK	78	38%
	Columbus, OH, USA	99	48%
	Beirut, Lebanon	30	14%
Study status	1st year	5	2%
	2nd year	72	35%
	3rd year	79	38%
Study mode	Full time	183	88%
	Part time	24	12%
Completed food safety training as part of degree	Yes	161	78%
	No	46	22%

**Table 2:** Food safety awareness according to dietetic students that received food safety education and that did not receive food safety education.

Food Safety Concepts	Total %	Food Safety Education %	No Food Safety Education %	Significant Differences	
**PATHOGEN AWARENESS**	***n*** **= 207**	***n*** **= 161**	***n*** **= 46**		
*E. coli*	94%	95%	46 (89)	*p* > 0.05	
*Salmonella*	96%	97%	46 (91)	*p* > 0.05	
*Listeria*	79%	83%	46 (63)	X^2^ (1, *n* = 207) = 8.710, *p* = 0.003, Cramer’s V = 0.205.	
*Campylobacter*	57%	68%	46 (15)	X^2^ (1, *n* = 207) = 41.059, *p* = 0.000, Cramer’s V = 0.445.	
*Staphylococcus*	72%	79%	46 (48)	X^2^ (1, *n* = 207) = 17.109, *p* = 0.000, Cramer’s V = 0.287	
*Clostridium*	64%	75%	46 (28)	X^2^ (1, *n* = 207) = 33.353, *p* = 0.000, Cramer’s V = 0.401	
**COOKING**	***n*** **= 197**	***n*** **= 154**	***n*** **= 43**		
Use a thermometer to check temperature	91%	90%	93%	*p* > 0.05	
**REFRIGERATION PRACTICES**	***n*** **= 190**	***n*** **= 152**	***n*** **= 38**		
Allow left-over cooked food to go cold before refrigerating	59%	66%	34%	X^2^ (1, *n* = 190) = 12.578, *p* = 0.000, Cramer’s V = 0.257	
Use a refrigerator thermometer to check operating temperature	67%	64%	79%	*p* > 0.05	
Awareness of recommended refrigeration temperatures	77%	89%	38%	X^2^ (1, *n* = 175) = 46.214, *p* = 0.000, Cramer’s V = 0.514	
**CROSS-CONTAMINATION RISKS**	***n*** **= 193**	***n*** **= 153**	***n*** **= 40**		
Failing to wash hands after handling raw meat before handling ready-to-eat food	95%	98%	85%	X^2^ (1, *n* = 193) = 12.127, *p* = 0.000, Cramer’s V = 0.251	
Storing raw meat above ready-to-eat in the refrigerator	90%	93%	75%	X^2^ (1, *n* = 193) = 11.638, *p* = 0.001, Cramer’s V = 0.246	
Using the same chopping/cutting board for raw and ready-to-eat food	94%	96%	85%	X^2^ (1, *n* = 193) = 6.674, *p* = 0.010, Cramer’s V = 0.186	
Washing raw poultry	59%	67%	25%	X^2^ (1, *n* = 193) = 23.401, *p*=0.000, Cramer’s V = 0.348	
Failing to clean a chopping board after cutting raw chicken before preparing salad	93%	95%	85%	X^2^ (1, *n* = 193) = 5.486, *p* = 0.019, Cramer’s V = −0.169	
After feeding or touching pets or animals	77%	83%	53%	X^2^ (1, *n* = 192) = 16.035, *p* = 0.000, Cramer’s V = 0.289	
**HANDLING LEFTOVERS**	***n*** **= 196**	***n*** **= 112**	***n*** **= 42**		
Reheat thoroughly only once	46%	74%	19%	X^2^ (1, *n* = 154) = 38.360, *p* = 0.000, Cramer’s V = 0.499	

**Table 3:** Food safety awareness according to dietetic students in United Kingdom, Lebanon and United States.

Food Safety Concepts	Total	Country			Significant Differences
		UK	Lebanon	USA	
**PATHOGEN AWARENESS**	***n*** **= 207**	***n*** **= 78**	***n*** **= 30**	***n*** **= 99**	
*E. coli*	94%	94%	93%	94%	*p* > 0.05
*Salmonella*	96%	97%	93%	95%	*p* > 0.05
*Listeria*	79%	82%	63%	81%	*p* > 0.05
*Campylobacter*	57%	71%	67%	42%	X^2^ (2, *n* = 207) = 15.476, *p* = 0.000, Cramer’s V = 0.273.
*Staphylococcus*	72%	86%	77%	60%	X^2^ (2, *n* = 207) = 15.346, *p* = 0.000, Cramer’s V = 0.272.
Clostridium	64%	74%	73%	54%	X^2^ (2, *n* = 207) = 9.496, *p* = 0.009, Cramer’s V = 0.214.
**COOKING**	***n*** **= 197**	***n*** **= 78**	***n*** **= 30**	***n*** **= 89**	
Use a thermometer to check temperature	91%	88%	83%	96%	*p* > 0.05
Ensure that the center is piping hot	40%	78%	47%	4%	X^2^ (2, *n* = 197) = 94.662, *p* = 0.000, Cramer’s V = 0.693.
Pierce thickest part to ensure juices run clear	45%	73%	37%	22%	X^2^ (2, *n* = 197) = 43.988, *p* = 0.000, Cramer’s V = 0.473
**REFRIGERATION PRACTICES**	***n*** **= 190**	***n*** **= 78**	***n*** **= 30**	***n*** **= 82**	
Check the operating temperature of the refrigerator	87%	79%	90%	93%	X^2^ (2, *n* = 190) = 6.403, *p* = 0.041, Cramer’s V = 0.041.
Allow left-over cooked food to go cold before refrigerating	59%	88%	60%	32%	X^2^ (2, *n* = 190) = 53.426, *p* = 0.000, Cramer’s V = 0.530
Refrigerate leftover foods immediately after cooking	22%	4%	20%	40%	X^2^ (2, *n* = 190) = 30.848, *p* = 0.000, Cramer’s V = 0.403
Use a refrigerator thermometer to check operating temperature	67%	53%	77%	77%	X^2^ (2, *n* = 190) = 12.171, *p* = 0.002, Cramer’s V = 0.253
Awareness of recommended refrigeration temperatures	77%	88%	97%	63%	X^2^ (2, *n* = 175) = 20.503, *p* = 0.000, Cramer’s V = 0.342
**STORAGE DURATION**	***n*** **= 195**	***n*** **= 78**	***n*** **= 30**	***n*** **= 87**	
Use by date	70%	81%	30%	75%	X^2^ (10, *n* = 195) = 61.505, *p* = 0.000, Cramer’s V = 0.394
Best before end date	10%	6%	40%	2%	
**HAND WASHING**	***n*** **= 192**	***n*** **= 78**	***n*** **= 30**	***n*** **= 84**	
Before commencing food preparation	96%	99%	80%	100%	X^2^ (2, *n* = 192) = 27.260, *p* = 0.000, Cramer’s V = 0.377
Before handling ready-to-eat foods	87%	79%	97%	90%	X2 (2, *n* = 192) = 7.259, *p* = 0.027, Cramer’s V = 0.194
After feeding or touching pets or animals	77%	97%	97%	51%	X2 (2, *n* = 192) = 56.684, *p* = 0.000, Cramer’s V = 0.543
**CROSS-CONTAMINATION RISKS**	***n*** **= 193**	***n*** **= 78**	***n* = 30**	***n* = 85**	
Failing to wash hands after handling raw meat before handling ready-to-eat food	95%	100%	93%	92%	X^2^ (2, *n* = 193) = 6.526, *p* = 0.038, Cramer’s V = 0.184
Washing raw poultry	59%	76%	47%	47%	X2 (2, *n* = 193) = 15.758, *p* = 0.000, Cramer’s V = 0.286
**HANDLING LEFTOVERS**	***n*** **= 196**	***n*** **= 78**	***n*** **= 30**	***n*** **= 88**	
Reheat thoroughly only once	46%	58%	70%	28%	X^2^ (1, *n* = 196) = 22.168, *p* = 0.000, Cramer’s V = 0.336
Warm up each time required	14%	4%	3%	27%	X^2^ (1, *n* = 193) = 22.002, *p* = 0.000, Cramer’s V = 0.335
**RECOMMENDED STORAGE DURATION**	***n*** **= 196**	***n*** **= 78**	***n*** **= 30**	***n*** **= 88**	
Cooked sliced meat/lunch meat	40%	37%	57%	38%	X^2^ (4, *n* = 196) = 16.989, *p* = 0.002, Cramer’s V = 0.208
Sliced cured meats/cold cuts	33%	24%	47%	36%	X^2^ (4, *n* = 196) = 19.692, *p* = 0.001, Cramer’s V = 0.224
Soft cheeses	29%	21%	27%	36%	*p* > 0.05
Smoked fish	34%	35%	20%	38%	X^2^ (4, *n* = 196) = 12.080, *p* = 0.017, Cramer’s V = 0.176
Pâté	34%	31%	43%	n/a	*p* > 0.05

**Table 4:** Perception of food safety risks to vulnerable populations and perception of food safety risks from different food preparation venues among students enrolled in dietetics programs in United Kingdom (*n* = 78), Lebanon (*n* = 29) and United States (*n* = 99).

Food Safety Perceptions	Low Quartile	Median	Upper Quartile	Country			Ranking (Weighted Average)
				UK	LEB	US	
**LIKELIHOOD OF BECOMING ILL AMONG VULNERABLE POPULATIONS**							
People with compromised immune system	10	10	10	9.59	9.38	9.67	9.60
People undergoing chemotherapy	9	10	10	9.37	9.39	9.53	9.45
People living with HIV	9	10	10	***	9.41	9.04	9.13
Older adults	8	9	10	8.81	8.76	9.11	8.95
Pregnant women	8	9	10	8.33	8.83	8.70	8.58
Young children	7	9	10	7.44	7.41	* 8.78	8.08
People with diabetes	5	6	8	***	***	6.24	6.24
General healthy population	3	5	6	4.43	4.79	4.75	4.63
**LIKELIHOOD OF FOOD FROM THE VENUE CAUSING FOODBORNE ILLNESS**							
Foods from take-away/fast-food restaurant	2	4	5.25	4.13	3.34	4.07	3.99
Food prepared by patients in their own home	4	5	7	5.35	4.71	5.72	5.43
Food prepared by caregivers of patients	4.5	6	7	5.71	6.11	5.88	5.82
Food provided in hospital foodservice	4.75	7	9	6.77	7.48	** 6.09	6.55

* The USA students ranked young children as significantly more likely to become ill with the foodborne disease, comparing to their peers in the UK and Lebanon (KW, *n* = 206, *p* < 0.05). ** The USA students ranked the foods prepared in hospital foodservice as significantly more likely to cause the foodborne disease, comparing to their peers in the UK and Lebanon (KW, *n* = 202, *p* < 0.05). *** Data not collected in all countries.

**Table 5:** Food safety attitudes among students enrolled in dietetics programs in the United Kingdom (*n* = 75), Lebanon (*n* = 29) and United States (*n* = 96).

Attitudes towards Food Safety Practices	Low Quartile	Median	Upper Quartile	Country			Ranking (Weighted Average)
				UK	LEB	US	
It is more important to implement food safety practices when patients immunosuppressed	4.00	4.00	4.00	4.34	4.38	4.40	4.37
A sealed pack of sliced cooked ham, 2 days past its ‘use-by’ date is still safe for vulnerable patients to eat	4.00	5.00	4.00	4.47	4.22	4.23	4.32
It is ok for people that have diarrhea to prepare food for vulnerable patients as long as they wash their hands first	4.00	5.00	4.00	4.55	3.36	4.23	4.22
It is safe for people in high-risk groups to eat soft cheese made from unpasteurized milk, like Brie or Camembert	4.00	4.00	4.00	4.32	4.17	4.17	4.22
Vulnerable patient groups are at no more risk of foodborne illness than the general population	4.00	5.00	4.00	4.10	4.10	4.09	4.10
It is acceptable to leave food at a lukewarm temperature for later consumption	4.00	4.00	4.00	4.03	4.10	4.01	4.03
Reheating food to a warm temperature is acceptable	3.00	4.00	3.00	4.22	3.72	3.24	3.67
The smell and taste of food are reliable indicators that food is safe to eat	3.00	4.00	3.00	3.61	3.38	3.70	3.62
Throwing food out that is only a few days past its ‘use-by’ date is wasteful	3.00	4.00	3.00	3.80	3.34	3.54	3.61
Food that has been opened for longer than two days is still safe for vulnerable patients to eat as long as it has been covered and kept in the fridge	3.00	4.00	3.00	3.68	3.76	3.34	3.53
It is safe for people in high-risk groups to drink juices and smoothies made with raw fruits and vegetables	2.00	3.00	2.00	2.36	3.32	3.19	2.90
Once packs of ready-to-eat food products have been opened, the ‘use-by’ date is no longer valid	2.00	3.00	2.00	3.07	2.76	2.75	2.87
Vulnerable patients can consume reheated leftover foods the following day	2.00	3.00	3.00	3.22	3.38	2.40	2.84

## Data Availability

All raw data supporting reported results is available from authors upon request.
